# Antitumor activity of MEK and PI3K inhibitors against malignant pleural mesothelioma cells *in vitro* and *in vivo*

**DOI:** 10.3892/ijo.2012.1462

**Published:** 2012-05-08

**Authors:** SEIGO MIYOSHI, HIRONOBU HAMADA, NAOHIKO HAMAGUCHI, AKI KATO, HITOSHI KATAYAMA, KAZUNORI IRIFUNE, RYOJI ITO, TATSUHIKO MIYAZAKI, TAKAFUMI OKURA, JITSUO HIGAKI

**Affiliations:** 1 Department of Integrated Medicine and Informatics, Ehime University Graduate School of Medicine, Ehime;; 2 Department of Health and Sports Medical Sciences, Graduate School of Health Sciences, Hiroshima University, Hiroshima;; 3 Department of Pathogenomics, Ehime University Graduate School of Medicine, Ehime, Japan

**Keywords:** malignant pleural mesothelioma, mitogen-activated protein kinase kinase inhibitor, phosphatidylinositol 3-kinase inhibitor, cell cycle, apoptosis, angiogenesis

## Abstract

Malignant pleural mesothelioma (MPM) is an aggressive malignancy for which there is no approved targeted therapy. We examined the therapeutic efficacy of the mitogen-activated protein kinase kinase (MEK) and phosphatidylinositol 3-kinase (PI3K) inhibitors against human MPM cell lines both *in vitro* and orthotopically inoculated into severe combined immunodeficient (SCID) mice. In addition, the molecular mechanisms of these agents were confirmed *in vitro* and *in vivo*. The MEK or the PI3K inhibitor suppressed MPM cell growth *in vitro* in a dose-dependent manner via induction of G1 cell cycle arrest and apoptosis. In addition, combined use of the MEK and PI3K inhibitors showed an additive or synergistic inhibitory effect on MPM cell growth compared to treatment with either individual drug. Treatment with MEK or PI3K inhibitor suppressed the production of thoracic tumors and pleural effusion and prolonged the survival time of EHMES-10 cell-bearing SCID mice. The combination therapy more effectively prolonged the survival time compared to treatment with either individual drug. Immunohistochemical and western blot analysis of thoracic tumors suggested that these agents induced cell cycle arrest, apoptosis and inhibition of tumor angiogenesis. Our results suggest that a combination of MEK and PI3K inhibitors is a promising therapeutic strategy for MPM.

## Introduction

Malignant pleural mesothelioma (MPM) is an aggressive neoplasm that arises from mesothelial cells. It was reported that asbestos, iron, and simian virus 40 were linked to the etiology of MPM (1–4). MPM was once considered a rare disease, but its incidence is increasing worldwide (5).

Current aggressive multimodality therapy for MPM (consisting of surgical resection, cytotoxic chemotherapy, and radiation) offers survival benefits for only a small subset of patients in early stages of the disease (6). Recently, the multi-targeted antifolate pemetrexed has been approved as the front-line agent in combination with cisplatin for the treatment of MPM (7). However, most of the patients relapsed within a year after starting treatments. Therefore, new and more effective therapies are necessary to improve the prognosis of this disease.

The mitogen-activated protein (MAP) kinase kinase (MEK)-extracellular signal-regulated kinase (ERK) pathway and the phosphatidylinositol 3-kinase (PI3K)-Akt pathway play critical roles in the regulation of cell proliferation, growth, differentiation and survival (8–10). These pathways are activated in many types of solid tumor models, including MPM (8,9,11–13). It was also reported that inhibition of these pathways affected the proliferation of MPM cell lines *in vitro*(14,15). However, no report has demonstrated growth-inhibitory effects of these agents on MPM cells *in vivo*.

In the present study, we examined whether the MEK or the PI3K inhibitor affected the growth of MPM cells *in vitro* and *in vivo*. Furthermore, we evaluated the possibility that combined use of the MEK inhibitor and the PI3K inhibitor might enhance MPM treatment.

## Materials and methods

### Cell cultures

The human mesothelioma cell line EHMES-10 was established from the pleural effusion of a patient with MPM in our institution (16,17). MSTO211H was purchased from the American Type Culture Collection (Manassas, VA, USA). These cell lines were cultured in RPMI-1640 medium (Nikken Bio Medical Laboratories, Kyoto, Japan) supplemented with 10% fetal bovine serum (Hyclone, Logan, UT, USA), penicillin (100 U/ml) and streptomycin (50 mg/ml) in a 37°C humidified incubator with 5% carbon dioxide.

### Reagents and inhibitors

MEK inhibitor U0126 and PI3K inhibitor LY294002 were purchased from LC Laboratories (Woburn, MA, USA). For *in vitro* experiments, these agents were dissolved in dimethylsulfoxide (DMSO) (Sigma-Aldrich Co., St. Louis, MO, USA) and were added to cells in medium with a final DMSO concentration of 1.0%. For *in vivo* studies, these agents were prepared as a suspension in a vehicle consisting of 40% DMSO in phosphate-buffered saline (PBS) (Wako Pure Chemical Industries, Osaka, Japan). Rabbit polyclonal antibodies against ERK1/2, phospho-ERK1/2, Akt, phospho-Akt, p27kip1, cyclin E, cyclin D1, p70S6K, phospho-p70S6K, S6, phospho-S6, p90 ribosomal S6 kinase (p90RSK), phospho-p90RSK, glycogen synthase kinase-3β (GSK3β), phospho-GSK3β, Bad, phospho-Bad, poly(ADP-ribose) polymerase (PARP), procaspase 3, hypoxia-inducible factor 1α (HIF1α), and β-actin were purchased from Cell Signaling Technology (Danvers, MA, USA). Rabbit polyclonal antibody against vascular endothelial growth factor (VEGF) was purchased from Millipore Co. (Tokyo, Japan). Mouse monoclonal antibody against CD31/platelet/endothelial cell adhesion molecule-1 was purchased from BD Pharmingen (Tokyo, Japan) for *in vivo* immunohistochemical study. Mouse monoclonal antibody against CD31 (PECAM-1) was purchased from Cell Signaling Technology for *in vivo* western blot analysis. Horseradish peroxidase conjugated goat anti-rabbit IgG and horse anti-mouse IgG were purchased from Cell Signaling Technology.

### Cell proliferation assay

The cell proliferation assay reagent WST-1 (4-[3-(4-lodophenyl)-2-(4-nitrophenyl)-2H-5-tetrazolio]-1,3-benzene disulfonate) (Roche Diagnostics GmbH, Mannheim, Germany) was used to assess the effect of U0126 or LY294002 on cell growth. MPM cells (1×10^4^ cells/well) were plated in 96-well plates (Nunc, Roskilde, Denmark) and were exposed to various concentrations of test agents dissolved in DMSO. Controls received DMSO vehicle at a concentration equal to that of drug treated cells. After drug treatment for 72 h, 10 *μ*l of WST-1 reagent were added to each well. Absorbance was measured at 450 nm with a reference wavelength at 690 nm by an E max precision microplate reader (Molecular Devices, Tokyo, Japan).

### Cell cycle analysis

MPM cells, treated with or without test agents for 24 h, were trypsinized and collected, and the cell nuclei were stained using the CycleTest Plus DNA Reagent Kit (Becton-Dickinson, San Jose, CA, USA). Cells were subjected to FACScan analysis, and cell cycle profiles were determined using ModFitLT software (Becton-Dickinson, San Diego, CA, USA). This analysis was carried out independently three times.

### DNA fragmentation assay

We examined DNA fragmentation to assess apoptosis in EHMES-10 or MSTO211H cells. Cells were treated with either U0126 or LY294002 or a combination of both for 24 h. DNA fragmentation was evaluated using the Cell Death Detection ELISA kit (Roche Molecular Biochemical, Indianapolis, IN, USA) as previously reported (18).

### Western blot analysis

Cultured cells were treated with lysis buffer [25 mM Tris-HCl (pH 7.5), 150 mM NaCl, 1% Triton X, 50 mM NaF and 1 mM Na_3_VO_4_] containing proteinase inhibitor cocktail (Roche Diagnostics GmbH). Tumor tissue samples were homogenized in lysis buffer. Insoluble materials were removed by centrifugation at 4°C for 15 min 15,000 × g. Protein concentration was determined using a Bio Rad Protein Assay Kit (Bio Rad Laboratories, Hercules, CA, USA).

Proteins were separated on 7.5 to 15% polyacrylamide gels (Bio Rad Laboratories). After electrophoresis, the protein was transferred to a nitrocellulose membrane and detected by immunoblotting using SNAP i.d. Protein Detection System (Millipore Co.) as previously described (19). This analysis was carried out independently three times.

### Experimental animals

Male severe combined immunodeficient (SCID) mice (six to eight weeks old) were obtained from Clea Japan (Osaka, Japan), fed autoclaved standard pellets and water, and maintained under specific pathogen-free conditions throughout this study. All of the protocols involving SCID mice were approved by the guidelines established by the Ehime University Committee on Animal Care and Use.

### Orthotopic implantation model

Cultured EHMES-10 cells were harvested, washed twice and re-suspended in PBS. The SCID mice were inoculated in the thoracic cavity with the tumor cells (3×10^6^ cells/mouse), as previously described (17,20). Seven days after inoculation, mice were randomized into eight groups (n=7 mice/group) to receive vehicle alone (DMSO + PBS), U0126 alone (20, 30 and 40 mg/kg), LY294002 alone (12.5, 25 and 50 mg/kg) and a combination of U0126 (30 mg/kg) and LY294002 (25 mg/kg). These agents were administrated intraperitoneally twice a week. Mice were sacrificed on day 30 after tumor cell inoculation. The tumor tissue was excised and weighed, and the volume of pleural effusion was measured. We also measured the body weights and serum levels of total protein (TP), blood urea nitrogen (BUN), creatinine (Cre), aspartate amino transferase (AST) and alanine aminotransferase (ALT), and evaluated the degree of dermatopathy as a measure of side effects.

### Immunohistochemistry

Paraffin-embedded tissues were subjected to immunohistochemistry with anti-phospho-ERK1/2 monoclonal antibody, phospho-Akt monoclonal antibody, or anti-p27kip1 monoclonal antibody. For *in situ* apoptosis detection, we used terminal deoxynucleotidyl transferase-mediated dUTP nick end labeling (TUNEL assay) with the *In situ* Apoptosis Detection Kit (Takara Biomedicals, Ohtsu, Japan). Frozen tissue sections were used for identification of endothelial cells using rat anti-mouse CD31/platelet/endothelial cell adhesion molecule-1 monoclonal antibody. Immunohistochemical procedures were performed using the Envision™ Systems (Dako, Glostrup, Denmark) method, as previously described (21). Phospho-ERK1/2- or phospho-Akt- or p27kip1-positive cells were visualized with Fuchsin^+^ substrate-chromogen (Dako). Antibodies against TUNEL assay or CD31 localization were detected using a peroxidase reaction with 3-diaminobenzidine (Dako).

### Statistical analysis

*In vitro* study data are presented as means ± SD, and were analyzed using ANOVA followed by Dunnett’s t-test. *In vivo* data were expressed as median values and ranges. The Mann-Whitney U test was used to compare groups. The Kaplan-Meier method was used to evaluate the survival analysis and comparisons were made using a log-rank test. Drug interactions were analyzed by the Chou and Talalay method using the CalcuSyn software program (version 2.0; Biosoft, Cambridge, UK). The combination index (CI) was simulated from each level of fractional affect. According to this method, a CI<0.3, 0.3–0.7, 0.7–0.9, 0.9–1.1, 1.1–1.45, 1.45–3.3 and >3.3 indicates highly synergistic, synergistic, moderate to slight synergistic, nearly additive, slight to moderate antagonistic, antagonistic and strong antagonistic, respectively. Differences between groups are considered statistically significant at P<0.05.

## Results

### Growth inhibition of MPM cells by U0126 and/or LY294002 treatment

**Figure 1:**
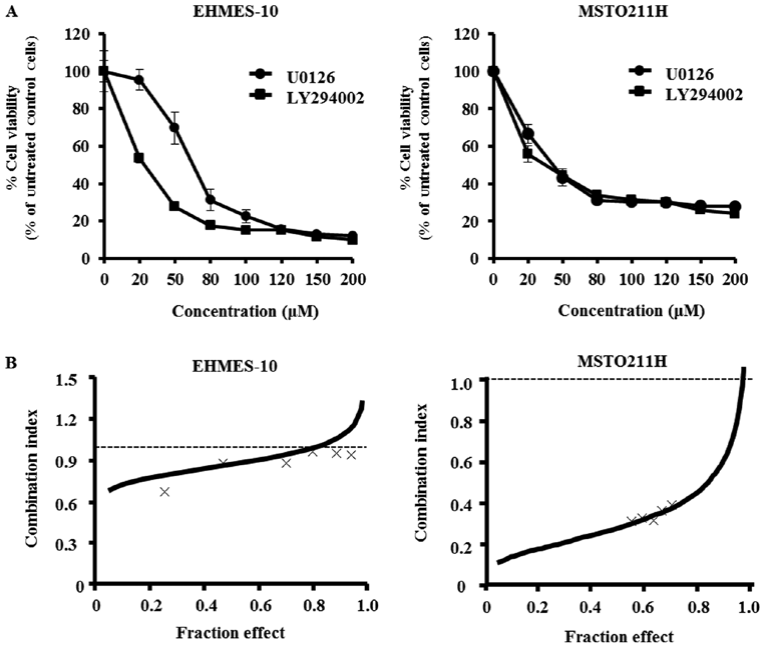
*In vitro* responses of EHMES-10 cells and MSTO211H cells to MEK and/or PI3K inhibitors. (A) The effects of U0126 or LY294002 on the proliferation of MPM cells. MPM cells were treated with U0126 or LY294002 for 72 h, and cell viability was determined with the WST-1 assay. (B) Analysis of the combined treatment of MPM cells with U0126 and LY294002. Inhibitors were used in combination in a fixed dose ratio for 72 h, and cell viability was assessed with the WST-1 assay. The fractional effect versus combination index (Fa-CI) curve was calculated with CalcuSyn software.

The effects of U0126 or LY294002 at concentrations ranging from 20 to 200 *μ*M on the proliferation of EHMES-10 or MSTO211H cells were determined with the WST-1 assay. Each agent inhibited MPM cell growth in a dose-dependent manner (Fig. 1A). The IC_50_ values for U0126 and LY294002 against EHMES-10 cells were 66.8 *μ*M and 20.7 *μ*M, respectively. Moreover, the IC_50_ values for U0126 and LY294002 against MSTO211H cells were 39.0 *μ*M and 29.9 *μ*M, respectively.

We evaluated the effect of combining treatments with U0126 and LY294002. The ratio of IC_50_ values for U0126 and LY294002 against EHMES-10 cells was approximately 3:1 while the ratio was 4:3 against MSTO211H cells. Therefore, the two MPM cell lines were exposed to varying concentrations of U0126 and LY294002 at fixed ratios of 3:1 or 4:3, as appropriate. Cell viability was then assessed by the WST-1 assay. The averaged CIs for EHMES-10 cells and MSTO211H cells were 1.017 and 0.54, which indicates a nearly additive effect and a synergistic effect, respectively (Fig. 1B).

### Induced G1 cell cycle arrest of MPM cells after treatment with U0126 and/or LY294002

**Figure 2:**
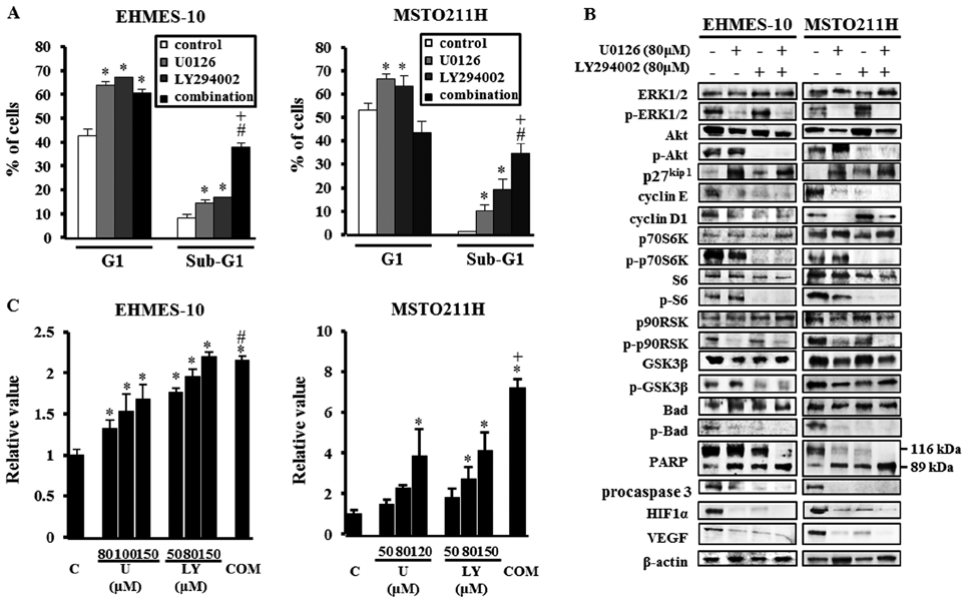
The mechanisms by which U0126 or LY294002 inhibited growth of EHMES-10 cells and MSTO211H cells *in vitro*. (A) The effects of U0126 and/or LY294002 on the cell cycle profile. After treatment with 80 *μ*M U0126 and/or 80 *μ*M LY294002 for 24 h, MPM cells were collected, fixed, strained with propidium iodide and analyzed by flow cytometry. Data shown are representative of three independent experiments. ^*^P<0.05, compared to control; ^#^P<0.01, compared to control; ^+^P<0.01, compared to individual drug. (B) Effects of U0126 and/or LY294002 on the expression of phospho-extracellular signal-regulated kinase (ERK)1/2 (p-ERK1/2), phospho-Akt (p-Akt), p27kip1, cyclin E, cyclin D1, phospho-p70S6K (p-p70S6K), phospho-S6 (p-S6), phospho-p90 ribosomal S6 kinase (p90RSK) (p-p90RSK), phospho-glycogen synthase kinase-3β (GSK3β) (p-GSK3β), phospho-Bad (p-Bad), poly (ADP-ribose) polymerase (PARP), procaspase 3, hypoxia-inducible factor 1α (HIF1α) and vascular endothelial growth factor (VEGF). Tumor cells were treated with or without U0126 (80 *μ*M), and/or LY294002 (80 *μ*M) for 24 h. Then, cells were lysed, and the indicated proteins were detected by immunoblotting. Data shown are representative of three independent experiments. (C) Cytoplasmic histone-associated DNA fragments determined by ELISA-based quantification. MPM cells were treated with U0126 or LY294002 or a combination of 80 *μ*M U0126 and 80 *μ*M LY294002 for 24 h. C, control; U, U0126; LY, LY294002; COM, combination of 80 *μ*M U0126 and 80 *μ*M LY294002; ^*^P<0.01, compared to control; ^#^P<0.01, compared to treatment with 80 *μ*M U0126 alone; ^+^P<0.01, compared to the individual drug.

To investigate the mechanisms of growth inhibition of MPM cells by U0126 or LY294002 treatment, we performed cell cycle analysis of EHMES-10 cells or MSTO211H cells treated with 80 *μ*M U0126 and/or 80 *μ*M LY294002. Treatment with U0126 or LY294002 for 24 h significantly increased the G1-phase populations compared to control in both MPM cell lines (all, P<0.05) (Fig. 2A). In addition, U0126 alone and LY294002 alone significantly increased the percentage of MPM cells in the sub-G1 phase, indicative of cell apoptosis, compared to control (all, P<0.05). Combining treatment with U0126 with that of LY294002 led to a significant increase in the sub-G1 phase population in both cell lines compared to control or individual drugs (all, P<0.01).

We also analyzed the expression of cell cycle regulatory proteins after treatment with U0126 and/or LY294002 in both EHMES-10 cells and MSTO211H cells. Both agents increased p27kip1 expression and decreased cyclin E expression in both cell lines (Fig. 2B). A decrease of cyclin D1 expression was observed in treatment with either U0126 or LY294002 in EHMES-10 cells, and following treatment with U0126 in MSTO211H cells.

### Induction of apoptosis by U0126 and/or LY294002 treatment

We assessed the ability of U0126 and LY294002 to induce apoptosis in MPM cells. DNA fragmentation analysis showed that treatment with U0126 or LY294002 alone induced apoptosis in EHMES-10 cells and in MSTO211H cells in a dose-dependent manner. Furthermore, the combined treatment with 80 *μ*M U0126 and 80 *μ*M LY294002 significantly increased the number of apoptotic cells compared to control and to treatment with U0126 alone in EHMES-10 cells (all, P<0.01) and compared to control and treatment with the individual drug in MSTO211H cells (all, P<0.01) (Fig. 2C). Western blot analysis showed that treatments with U0126 and/or LY294002 increased the level of 89 kDa cleaved PARP and decreased the levels of phospho-Bad and procaspase 3 in both cell lines (Fig. 2B).

### Signaling alterations induced by treatment with U0126 and/or LY294002

To investigate the effects of U0126 and LY294002 on intercellular signaling, MPM cells were treated with U0126 or LY294002 or a combination of both. As shown in Fig. 2B, U0126 blocked the phosphorylation of ERK1/2 and p90RSK, and decreased HIF1α and VEGF expression in EHMES-10 cells. On the other hand, treatment with LY294002 suppressed the phosphorylation of Akt, p70S6K, S6 and GSK3β, and inhibited HIF1α and VEGF expression in EHMES-10 cells. Use of the combination treatment inhibited the phosphorylation of all of the above proteins and decreased the level of HIF1α and VEGF in EHMES-10 cells. Signaling alterations in MSTO211H cells tended to be similar to those in EHMES-10 cells, except for phospho-GSK3β alteration.

### Antitumor activity of U0126 and/or LY294002 in EHMES-10 cell xenografts

**Figure 3:**
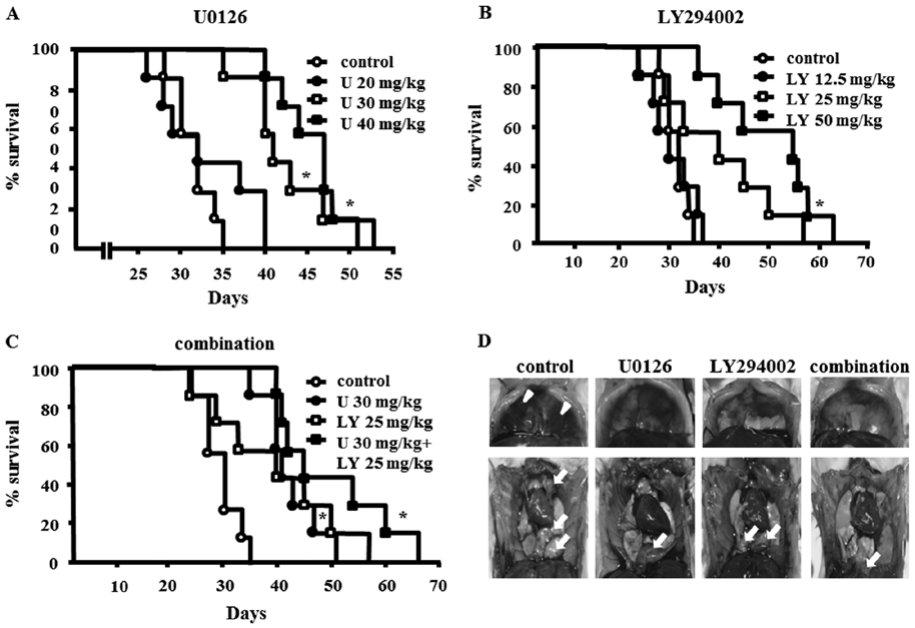
Effects of U0126 or LY294002 or the combination of U1026 and LY294002 on severe combined immunodeficiency (SCID) mice bearing EHMES-10 cells. (A–C) Survival times of EHMES-10 cell-bearing SCID mice treated with U0126, LY294002, or the combination of U0126 and LY294002. EHMES-10 cells (3×10^6^) were inoculated into the thoracic cavity of SCID mice. Seven days after inoculation, SCID mice were randomized into eight groups (n=7 mice/group) to receive vehicle (DMSO + PBS), U0126 (20, 30 and 40 mg/kg), or LY294002 (12.5, 25 and 50 mg/kg), or a combination of U0126 (30 mg/kg) and LY294002 (25 mg/kg). U, U0126; LY, LY294002; ^*^P<0.01, compared to control. (D) Formation of thoracic tumors and pleural effusion by EHMES-10 cells with or without test agents. Mice were sacrificed on day 30 and thoracic tumors and pleural effusions were evaluated. Arrowheads, pleural effusions; arrows, thoracic tumors.

To assess the *in vivo* therapeutic efficacy of U0126 and/or LY294002, SCID mice bearing EHMES-10 xenografts were treated with vehicle, U0126, LY294002, or a combination of U0126 and LY294002 as described in Materials and methods. Administration of 30 or 40 mg/kg of U0126, or 50 mg/kg of LY294002, or use of combined therapy with 30 mg/kg of U0126 and 25 mg/kg of LY294002 significantly prolonged the survival time of EHMES-10 cell-bearing SCID mice compared to the control group (all, P<0.01) (Fig. 3A–C). The combination therapy more effectively prolonged the survival time compared to treatment with either individual drug, although statistical significance was not obtained (Fig. 3C).

We also evaluated the effect of U0126 and/or LY294002 on the production of thoracic tumors and pleural effusion in EHMES-10 cell-bearing SCID mice (Fig. 3D, Table I). U0126 and/or LY294002 significantly inhibited tumor growth and pleural effusion production compared to control (all, P<0.05). The combination therapy more effectively inhibited tumor growth compared to treatments with individual drugs, although statistical significance was not obtained.

### Immunohistochemical staining and western blot analysis to clarify the antitumor mechanisms of U0126 and/or LY294002 in vivo

**Figure 4:**
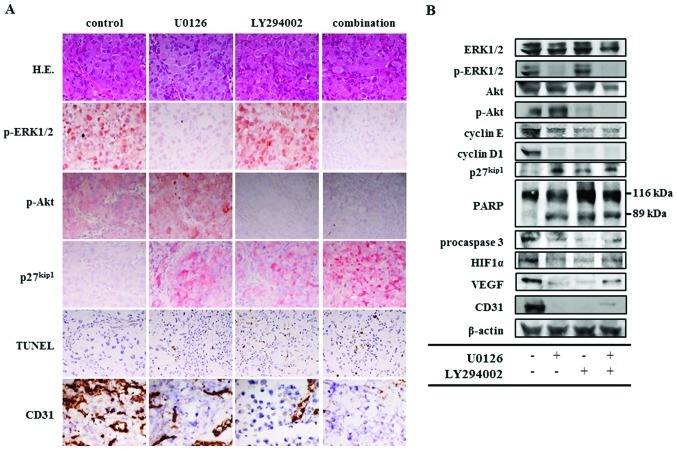
Histological and western blot analysis of thoracic tumors produced by EHMES-10 cells. SCID mice bearing EHMES-10 cells were treated with 30 mg/kg U0126, 25 mg/kg LY294002 and a combination of 30 mg/kg U0126 and 25 mg/kg LY294002. Mice were sacrificed on day 30 after tumor cell inoculation. (A) Thoracic tumors were analyzed by H&E and immunohistochemistry of phospho-extracellular signal-regulated kinase (ERK)1/2 (p-ERK1/2), phospho-Akt (p-Akt), p27kip1, TUNEL and CD31. Magnification, ×400. (B) Western blots analysis showing modulation of phospho-extracellular signal-regulated kinase (ERK)1/2 (p-ERK1/2), phospho-Akt (p-Akt), cyclin E, cyclin D1, p27kip1, poly(ADP-ribose) polymerase (PARP), procaspase 3, hypoxia-inducible factor 1α (HIF1α), vascular endothelial growth factor (VEGF) and CD31 after treatment with U0126 and/or LY294002.

Immunohistochemical analysis showed that treatment with U0126 or with LY294002 reduced phospho-ERK1/2-positive tumor cells or phospho-Akt-positive tumor cells, respectively (Fig. 4A). Furthermore, treatment with an individual drug increased the number of p27kip1-positive tumor cells and TUNEL assay-positive tumor cells, and decreased the number of CD31-positive endothelial cells. The combination therapy group showed decreased phospho-ERK1/2 and phospho-Akt activities and CD31-positive endothelial cells, and increased p27kip1-positive tumor cells and TUNEL assay-positive tumor cells. The effect of the inhibitors delivered in combination was more pronounced than the drugs applied individually in the analyses of p27kip1 and TUNEL assay.

Western blot analysis showed that treatment with U0126, LY294002 and a combination of these agents inhibited ERK1/2 phosphorylation, Akt phosphorylation, and the phosphorylation of ERK1/2 and Akt, respectively (Fig. 4B). In treatment with U0126, LY294002 and the combination, we observed inhibited expression of cyclin E, cyclin D1, procaspase 3, HIF1α, VEGF, and CD31, and increased expression of p27kip1 and 89 kDa cleaved PARP.

### Side effects of treatment with U0126 and/or LY294002

To examine the side effects of treatment with U0126 and/or LY294002, the body weights and serum levels of TP, BUN, Cre, AST and ALT were determined at the end of therapy on day 30 (Table II). Dermatopathy was also evaluated during the treatment with these agents. Side effects were not observed after the administration of these agents.

## Discussion

The Ras pathway is one of the most frequently deregulated pathways in cancer (22). Ras signals through multiple effector pathways, including the RAF/MEK/ERK and PI3K/Akt signaling cascades. A previous study reported that these pathways were frequently activated in MPM (13). Therefore, downregulation of these pathways might contribute to the inhibition of tumor development and progression. In this study, we showed that treatment with MEK and PI3K inhibitors, U0126 and LY294002, inhibited MPM cell growth via cell cycle arrest, apoptosis, and inhibition of tumor angiogenesis *in vitro* and *in vivo*. In addition, each drug prolonged the survival time of SCID mice bearing EHMES-10 cells. When drugs were applied in combination, survival times were longer than those achieved with the individual treatments.

Treatment with a MEK inhibitor or a PI3K inhibitor has been shown to inhibit the growth of many types of cancer cells, including MPM cell lines, via induction of cell cycle arrest and apoptosis (14,15,23–25). In the present study, MEK and PI3K inhibitors suppressed growth of MPM cells in a dose-dependent manner. Flow cytometric analysis showed that the treatment with MEK or PI3K inhibitors achieved G1 cell cycle arrest of MPM cells. DNA fragmentation analysis showed apoptosis of MPM cells following treatment with these agents. It was reported that treatment of MPM with a MEK inhibitor induced p27kip1 upregulation (26). A PI3K inhibitor induced p27kip1 upregulation and inhibition of phosphorylation of p70S6K and S6 (14,26). The present study showed similar results. In addition, treatment of MPM cells with a MEK inhibitor downregulated cyclin E and cyclin D1, and inhibited phosphorylation of p90RSK and Bad. Inhibition of cyclin E and phospho-Bad expression was observed in treatment with a PI3K inhibitor for MPM cells. Treatment of EHMES-10 cells with a PI3K inhibitor reduced cyclin D1 and phospho-GSK3β expression.

Our study demonstrated the efficacy and the mechanisms of action of a MEK inhibitor and a PI3K inhibitor for MPM not only *in vitro* but also in *in vivo* experiments. All previous reports of MEK inhibitors and PI3K inhibitors for MPM cells have been limited to showing the inhibitory effects and mechanisms *in vitro*(14,26). Our study showed that treatment with a MEK inhibitor or a PI3K inhibitor prolonged the survival time of EHMES-10 cells-bearing SCID mice. Tumor weight and pleural effusion at day 30 were reduced by these treatments. Furthermore, the immunohistochemical and western blot analyses of thoracic tumors suggested that the MEK and the PI3K inhibitors induced cell cycle arrest and cell apoptosis, which was compatible with the results of the *in vitro* study.

Treatments with the MEK inhibitor or the PI3K inhibitor might be associated with inhibition of angiogenesis in MPM cells. More than 60% of patients with MPM commonly present with a pleural effusion associated with breathlessness, often accompanied by chest wall pain, which compromises their quality of life (27). Angiogenesis has significant effects on the development of a pleural effusion and ascites (28,29). It was also reported that treatment with a MEK or a PI3K inhibitor suppressed proangiogenic cytokine production in melanoma and MPM cells (15,23). Our study showed that these agents significantly inhibited pleural effusion production and CD31 protein expression and decreased CD31-positive endothelial cells compared to controls. In addition, western blot analysis showed that treatment of these agents decreased the expression of HIF1α and VEGF, both of which play an essential role in tumor angiogenesis and progression, *in vitro* and *in vivo*.

Combination therapy with the MEK and PI3K inhibitors might be more rational than an individual drug for MPM. It was reported that antitumor activity of a MEK inhibitor or a PI3K inhibitor induces activation of the other pathway (30). Moreover, several reports demonstrated that inhibition of both cascades results in greater antitumor activity (15,23). In the present study, the combination therapy with MEK and PI3K inhibitors was also more effective compared to that of individual drugs both *in vitro* and *in vivo*.

Treatment with a MEK inhibitor and a PI3K inhibitor might be well tolerated. For example, treatment with the MEK inhibitor CI-1040 caused only mild or moderate toxicities such as diarrhea, nausea, asthenia, rash, and anorexia in patients with advanced non-small cell lung, breast, colon, and pancreatic cancers (31). In addition, Hu *et al* reported that daily intraperitoneal administration of LY294002 at a dose of 100 mg/kg caused body weight loss and dry skin in mice with ovarian cancer (32). However, in the following study, side effects were not shown by reduction of LY294002 administration to three days per week (33). In the present study, side effects were not observed using a combination of MEK and PI3K inhibitors.

In conclusion, our study demonstrates that in MPM cells our selected MEK and PI3K inhibitors functioned via cell cycle arrest, induction of apoptosis, and inhibition of tumor angiogenesis, both *in vivo* and *in vitro*. In addition, combining the MEK inhibitor with the PI3K inhibitor had additive or synergistic effects *in vitro*. Combination therapy with MEK and PI3K inhibitors may represent a promising novel therapeutic strategy in the treatment of MPM.

## Figures and Tables

**Table I:** Effects of U0126, LY294002 and combination therapy on thoracic tumor and pleural effusion produced by MPM cells in SCID mice.

	Thoracic tumor		Pleural effusion	
	Incidence	Weight (mg)	Incidence	Volume (*μ*l)
Control	5/5	487.7 (162.8–907.1)	5/5	150 (50.0–280.0)
U0126				
30 mg/kg	4/5	31.3 (0–59.2)^a^	1/5	0 (0–100)^a^
40 mg/kg	3/5	14.6 (0–191.3)^a^	0/5	0 (−)^a^
LY294002				
25 mg/kg	3/5	69.1 (0–481.6)^a^	1/5	0 (0–10)^a^
50 mg/kg	2/5	0 (0–41)^a^	0/5	0 (−)^a^
Combination	3/5	7.5 (0–8.6)^a^	0/5	0 (−)^a^

EHMES-10 cells were inoculated into thoracic cavity of severe combined immunodeficiency mice on day 0, and the mice were treated with U0126 (30 or 40 mg/kg) or LY294002 (25 or 50 mg/kg) or combination with 30 mg/kg U0126 and 25 mg/kg LY294002 i.p. on twice/week. Mice were sacrificed on day 30 and thoracic tumor and pleural effusion were evaluated. Data are median values (ranges). U, U0126; LY, LY294002;

a P<0.05 compared to control.

**Table II:** Side effects of treatment with U0126 or LY294002 or combination therapy after 30 days.

	Weight (g)	TP (g/dl)	BUN (mg/dl)	Cre (mg/dl)	AST (IU/l)	ALT (IU/l)
Control	24.8 (19.7–25.7)	4.6 (4.5–4.8)	17.1 (14.8–18.5)	0.15 (0.10–0.21)	55 (48–66)	36.5 (23–42)
U30 mg/kg	24.1 (23.5–27.2)	4.3 (4.0–5.0)	22.2 (15.4–24.0)	0.10 (0.08–0.15)	48 (44–64)	30 (20–60)
U40 mg/kg	24.9 (23.3–25.9)	5.0 (4.4–6.6)	19.1 (15.3–22.1)	0.07 (0.05–0.12)^a^	75 (53–111)	45 (31–80)
LY25 mg/kg	25.4 (24.4–26.3)	4.3 (3.9–5.6)	20.2 (17.9–23.0)	0.11 (0.06–0.20)	46 (37–117)	19 (15–72)
LY50 mg/kg	24.4 (22.7–26.3)	4.4 (3.9–5.6)	21.0 (16.4–23.2)	0.09 (0.07–0.12)^a^	51 (46–92)	25 (16–42)
Combination	23.5 (22.5–23.7)	4.9 (4.6–5.0)	24.9 (18.0–25.7)	0.06 (0.04–0.07)^a^	63.5 (62–102)	34 (30–79)

Mice were treated with U0126 (30 or 40 mg/kg) or LY294002 (25 or 50 mg/kg) or combination with 30 mg/kg U0126 and 25 mg/kg LY294002 i.p. twice/week, and the body weights, serum levels of total protein (TP), blood urea nitrogen (BUN), creatinine (Cre), aspartate amino transferase (AST) and alanine aminotransferase (ALT) were evaluated at the end of therapy on day 30. Data are median values (ranges). U, U0126; LY, LY294002;

a P<0.05 compared to control.
